# A-to-I edited miR-154-p13-5p inhibited cell proliferation and migration and induced apoptosis by targeting LIX1L in the bladder cancer

**DOI:** 10.7150/jca.93388

**Published:** 2024-05-13

**Authors:** Zhengxiang Hu, Chunhui Liu, Zujun Mei, Xinlei Wang, Yuyang Ma, Xing Liu, Hao Xu, Gaochuan Fang, Xinyu Liu, Rui Li, Jie Wang, Zhenduo Shi, Conghui Han

**Affiliations:** 1 Postgraduate Training Base of Jinzhou Medical University in The Central Hospital of Xuzhou, Jinzhou, Liaoning 121013, China.; 2 Department of Urology, Xuzhou Central Hospital, Xuzhou, Jiangsu 221006, China.; 3 Xuzhou Clinical College of Xuzhou Medical University, Xuzhou 221004, China.; 4 Department of Emergency, Jingzhou Central Hospital, Jingzhou, Hubei 434000, China.; 5 Graduate School of Bengbu Medical College, Bengbu, Anhui 233060, China.; 6 School of Life Sciences, Jiangsu Normal University, Xuzhou, Jiangsu 221116, China.; 7 Central Laboratory, Xuzhou Central Hospital, Jiangsu 221006, China.

**Keywords:** bladder cancer, miRNA editing, miR-154-p13-5p, LIX1L

## Abstract

With the advancement of RNA sequencing technology, there has been a drive to uncover and elucidate the pivotal role of A-to-I RNA editing events in tumorigenesis. However, A-to-I miRNA editing events have been clearly identified in bladder cancer, the molecular mechanisms underlying their role in bladder cancer remain unclear. In our investigation, we observed a notable under-expression of edited miR-154-p13-5p in bladder cancer (BC) tissues, in contrast to normal counterparts. Remarkably, heightened expression levels of edited miR-154-p13-5p correlated with improved survival outcomes. To assess the impact of modified miR-154-p13-5p, we conducted a string of cell phenotype assays through transfection of the corresponding miRNAs or siRNAs. The results unequivocally demonstrate that edited miR-154-p13-5p exerts a substantial inhibitory influence on proliferation, migration, and induces apoptosis by specifically targeting LIX1L in bladder cancer. Moreover, we observed that the editing of miR-154-p13-5p or LIX1L-siRNAs inhibits the expression of LIX1L, thereby suppressing EMT-related proteins and cell cycle protein CDK2. Simultaneously, an upregulation in the expression levels of Caspase-3 and Cleaved Caspase-3 were also detected. Our research findings suggest that the upregulation of edited miR-154-p13-5p could potentially enhance the prognosis of bladder cancer, thereby presenting molecular biology-based therapeutic strategies.

## Introduction

Bladder cancer (BC) ranks as the tenth most common malignant tumor worldwide. Approximately 573,278 individuals are diagnosed with bladder cancer each year, with the majority of cases occurring in male patients 1. Pathological types of BC encompass bladder adenocarcinoma bladder squamous cell carcinoma (BSCC), bladder urothelial carcinoma (BUC), and other rare variants, with BUC being the most common 2. BUC further segregates into non-muscle-invasive bladder carcinoma (NMIBC) and muscle-invasive bladder carcinoma (MIBC), predicated upon the degree of tumor invasiveness 3, 4. Despite significant advancements in surgical and chemotherapeutic approaches for treating bladder cancer, the substantial recurrence and metastasis rates post-surgery persist as challenging issues 5. Consequently, exploring the molecular mechanisms to improve the prognosis of bladder cancer patients has become a research hotspot around the world.

A-to-I RNA editing event is referred as a modification process, wherein adenosine (A) is deaminated to inosine (I) in the nucleotide sequence of transcripts 6, 7. The integrated development of computer science and bioinformatics has facilitated the identification of approximately 1.6 million A-to-I editing sites in the human genome 8. Although a relatively fewer fraction of A-to-I RNA editing sites resides within coding regions, these modifications directly translate into alterations within the amino acid sequences in this region, thereby expanding the diversity of the human proteome 9, 10. A-to-I editing primarily manifests within non-coding transcripts, particularly in Untranslated Regions (UTRs), long non-coding RNAs (LncRNAs), and microRNAs (miRNAs) 11-13.

MiRNAs represent the category of non-coding, single-stranded RNA molecules, typically comprising around 21-23 nucleotides 14. MiRNAs execute their regulatory influence by selectively targeting the 3'UTR of downstream mRNAs, thereby impinging on mRNA stability and translation suppression 15. Under the guidance of RNA Polymerase II, miRNA is initially transcribed to produce a primary transcript known as pri-miRNA 16. Following recognition and cleavage of pri-miRNA by Drosha-DGCR8 into a hairpin-shaped pre-miRNA, the pre-miRNA is then transported to the cytoplasm through the Exportin-5 complex 17. In the cytoplasm, the pre-miRNA is cleaved by the Dicer to form a mature miRNA duplex. Subsequently, the mature miRNA is loaded onto the Argonaute (AGO) protein, forming the miRNA-induced silencing complex (miRISC). The AGO protein selectively chooses between the two strands of the miRNA duplex, leading to the degradation of one strand, while the other strand initiates its regulatory function 18.

As of now, growing evidence has highlighted that pri-miRNAs are subjected to A-to-I RNA editing. In human brain tissue, researchers successfully utilized PCR technology to discover that 49 out of 209 pri-miRNAs have A-to-I RNA editing sites 19. Moreover, it is worth noting that Next-Generation Sequencing (NGS) has enabled the identification of A-to-I editing sites in select mature miRNAs 20-22. A-to-I edited miRNAs can alter their specific targets due to changes in individual sites, thereby influencing their biological significance. For instance, in melanoma, miR-455-5p progressed melanoma metastasis by targeting CPEB1, a well-established tumor suppressor. Intriguingly, the edited form of miR-455-5p loses its ability to target CPEB1, resulting in the inability to inhibit its expression 23. Similarly, A-to-I RNA editing manifests at the fourth nucleotide of the miR-376a-5p seed region, leading to the redirecting of phosphoribose pyrophosphate synthetase 1 (PRPS1) and the consequent suppression of its expression. PRPS1 is implicated in purine metabolism and uric acid synthesis pathways, and elevated expression of PRPS1 is associated with conditions such as gout and neurodevelopmental disorders characterized by hyperuricemia in humans 24. These findings underscore the profound role of A-to-I RNA editing in normal metabolic processes and tumor progression.

In this study, our methodology investigated the differential expression levels of edited miR-154-p13-5p in BC and its correlation with patients' survival time. We characterized the role of edited miR-154-p13-5p and further identified key target through mRNA-Seq screening. We analyzed the potential regulatory pathways of edited miR-154-p13-5p to gain deeper insights into its regulatory mechanisms. These revelations not only deepen our comprehension of the intricate pathogenesis and evolution of BC but also provide potential avenues for targeted therapeutic interventions.

## Materials and methods

### Bioinformatics analysis

Transcriptome data was obtained from the YM500 database, which included TCGA-sequencing data for research purposes 25. We acquired the miRNA-seq data, consisting of 19 samples of normal bladder tissue and 179 samples of bladder cancer tissue, focusing on edited miR-154-p13-5p. Additionally, we extracted relevant clinical information to evaluate whether high or low editing expression correlated with Progression-Free Survival (PFS) improvement using Kaplan-Meier curves.

### Tissue samples collection

Tissue sample collection at the Department of Urology, Xuzhou Central Hospital, Jiangsu Province, China, was conducted from December 2021 to July 2023. We obtained consent from 6 bladder cancer patients and collected relevant tissues during laparoscopic radical cystectomy (LRC). Following surgery, the tissues were cleaned with 0.9% physiological saline and placed in cryopreservation tubes. They were immediately stored in a liquid nitrogen environment until they were used for qPCR testing. Table 1 summarizes a total of 12 tissue samples, comprising 6 cases of bladder cancer tissue and 6 cases of para-cancerous tissues. The study protocol received approval from the Ethics Committee of Xuzhou Central Hospital.

### Cell culture

The human bladder cancer cell lines T24, 5637, and BIU-87, as well as the human normal bladder cell line SV-HUC-1, were obtained from the American Type Culture Collection (ATCC). T24, 5637 and BIU-87 cells were cultured in RPMI1640 medium (Gibco), while SV-HUC-1 cells were cultured in Ham's F-12K (Procell). In both culture media preparation processes, 10% fetal bovine serum (Wisent Catalog No. 086-150), 100 IU/mL penicillin, and 100 µg/mL streptomycin (Gibco) were added. All cells were incubated in a culture chamber at 37°C, 5% CO2, and under conditions of relative humidity.

### Transfection

The miRNA mimics were obtained from Ribo Biotechnology Company (Guangzhou, China) and included the following: mimics-NC, miR-154-p13-5p, and edited miR-154-p13-5p. SiRNAs were sourced from General Biotechnology Company (Anhui, China) and consisted of three LIX1L-specific siRNAs and siRNA-NC. The sequences of the miRNAs and siRNAs used are presented in Table 2. Following the manufacturer's instructions, we separately incubated miRNAs or siRNAs with transfection reagents (Lipofectamine™ RNAiMAX, Thermo Fisher Scientific) in serum-free medium (Opti-MEM I, Thermo Fisher Scientific) for 5 minutes. Subsequently, we mixed them for 20 minutes before adding them to a six-well plate with cell confluence ranging from 70% to 80%.

### Cell proliferation assay

After transfection, the cells were seeded into 96-well plates at a density of 5000 cells/well. Distinct time points at 0, 24, 48, and 72 hours were designated as observation times. At these specified time points, 100 µL of an incubation solution, comprising 90 µL of complete medium and 10 µL of CCK-8 reagent (Vicmed, China), was added to each well of the 96-well plate. Following 1 hour of incubation in a light-protected environment, the optical density (OD) value corresponding to each well was measured at 450 nm using a microplate reader (Infinite® 200 PRO, Tecan).

### Cell apoptosis assay

After digesting the cells with EDTA-free trypsin (Beyotime, China) for harvesting, the resulting cell suspension underwent centrifugation at 1000 RPM, 4°C for 5 minutes to eliminate the supernatant. After washing with PBS, 500 µL of binding buffer was introduced to suspend the cells. After staining the cells with 5 µL Annexin V-FITC and 5 µL Propidium iodide (BD, USA), apoptosis was detected using flow cytometry (CytoFLEK, Beckman).

### Flow cytometry

10^5 cells were seeded in a 10 cm cell culture dish, followed by overnight incubation in a 5% carbon dioxide incubator. After 48 hours of transfection, harvested cells were resuspended in 800 µL pre-cooled 70% ethanol to prepare a single-cell suspension. Following overnight fixation at 4°C, cells were resuspended in 200 µL PBS. A mixture containing 10 µL RNase and 10 µL propidium iodide (FxCycle™ PI/RNase, Invitrogen) was added, and flow cytometry was employed for cell cycle distribution analysis.

### Wounding healing assay

5×10^4 cells were seeded within a culture-insert gap (ibidi, Germany) and incubated overnight. Upon removal of the insert, this action created a 500 µm gap on both sides of the cells. Subsequently, following various treatments, we acquired images at 0 hours and 24 hours using a phase-contrast microscope (Olympus Corporation, Japan) and quantified cell migration distance using ImageJ software (NIH, MD).

### mRNA sequencing and data analysis

Total RNAs were extracted from T24 cells transfected with the corresponding miRNA using TRIzol Reagent (Invitrogen, Cat. No. 15596026). For stranded RNA sequencing library preparation, 2 µg of total RNAs were used with the KCTM Stranded mRNA Library Prep Kit for Illumina® (Catalog No. DR08402, Wuhan Seqhealth Co., Ltd, China) following the manufacturer's instructions. PCR products ranging from 200 to 500 bps were enriched, quantified, and sequenced on the DNBSEQ-T7 sequencer (MGI Tech Co., Ltd, China) using the PE150 model.

Raw sequencing data underwent initial filtering using Trimmomatic (version 0.36) to discard low-quality reads and trim adaptor sequences. Clean data were then mapped to the reference genome of *Homo sapiens* from the GRCh38/hg38 reference using STRA software (version 2.5.3a) with default parameters. Reads mapped to exon regions of each gene were counted using featureCounts (Subread-1.5.1; Bioconductor), and RPKMs (Reads Per Kilobase Million) were calculated. Differential gene expression analysis was performed using the edgeR package (version 3.12.1) with a p-value cutoff of 0.05 and a fold-change cutoff of 2. Gene Set Enrichment Analysis (GSEA) was conducted using javaGSEA (version 4.2.3).

### Quantitative real time polymerase chain reaction (qRT-PCR)

Cellular RNA extraction was performed utilizing the RNA-easy Isolation Reagent (R701-01, Vazyme). For cDNA synthesis, MiRNA 1st Strand cDNA Synthesis Kit (MR101, Vazyme) was employed following the Stem-loop Method. To evaluate edited miR-154-p13-5p expression, PCR assays were completed by using the MiRNA Universal SYBR qPCR Master Mix (MQ101, Vazyme). In the case of LIX1L expression analysis, we completed reverse transcription using the HiScript II Q RT SuperMix for qPCR (R223, Vazyme), and qPCR assays were executed employing the ChamQ SYBR qPCR Master Mix (Q331, Vazyme). Relative data for each sample were computed through QuantStudio 3 (Thermo Fisher Scientific, USA) and 2-ΔΔCT formula 26.

The primer sequences employed were as follows: For edited miR-154-P13-5p and U6, Bulge-loop™ miRNA qRT-PCR Primer Sets (comprising one RT primer and a pair of qPCR primers) were designed by RiboBio (Guangzhou, China). For LIX1L and GAPDH, primer sequences were provided by Sangon Biotech (Shanghai, China). The sequences were as followed: LIX1L-Forward: 5'-CGCCGCTGCTCCTGTCTG-3'/LIX1L-Reverse: 5'-TGCCTCCACCACATTCACTG-3'; GAPDH-Forward: 5-CAGGAGGCATTGCTGATAT-3'/GAPDH-Reverse: 5'-GAAGGCTGGGGCTCATTT-3'.

### Luciferase reporter assay

Based on the sequence of edited miR-154-p13-5P, two binding regions were identified in the 3'UTR region of LIX1L, denoted as WT-LIX1L-1 and WT-LIX1L-2. These binding sites were individually cloned into the pmirGlo vector, resulting in pmirGlo-NC, pmirGlo-LIX1L-1, and pmirGlo-LIX1L-2 constructs. We conducted co-transfection experiments with the respective plasmids and miRNAs in T24 cells using GP-Transfect-Mate (G04008, Genepharma). Following transfection, the cells were sent for incubation at 37°C for 48 hours, and cellular contents were subsequently extracted using cell lysis buffer (Gibco). To measure luciferase activity, 30 µL of firefly luciferase and 30 µL of renilla luciferase substrate (Promega) were successively added, and the activity was quantified.

### Western blot

Cellular lysis was accomplished using lysis buffer (P0013B, Beyotime) containing phenylmethylsulfonyl fluorid (ST505, Beyotime) and Protease Inhibitor Cocktail (CW2200S, CWBIO). The total cellular protein concentration was quantified using the BCA protein concentration assay kit (P0010, Beyotime). Subsequently, SDS-PAGE was performed, and the PVDF membrane was incubated overnight with specific antibodies. For blot detection, a HRP-conjugated anti-Rabbit antibody (SA00001-2, Proteintech) served as the secondary antibody. The internal control was probed with an anti-β-actin antibody (66009, PTG). ECL luminescence reagent (P0018S, Beyotime) was employed to visualize the protein bands. Protein band densities were quantified and analyzed using AIWBwellTM (ServiceBio, China).

### Statistical analysis

GraphPad 8.0 (California, USA), Image J (National Institutes of Health, NIH) and Adobe Illustrator (Adobe, USA) software were used for statistical analysis and graphing. The two groups of data conforming to normal distribution were statistically analyzed by t-test; the two groups of data conforming to skewed distribution were statistically analyzed by Mann-Whitney U test; and the analysis between multiple groups was performed by one-way analysis of variance (ANOVA). P-value <0.05 indicated statistical significance.

## Results

### Edited miR-154-p13-5p expression was down-regulated in BC

**Figure 1:**
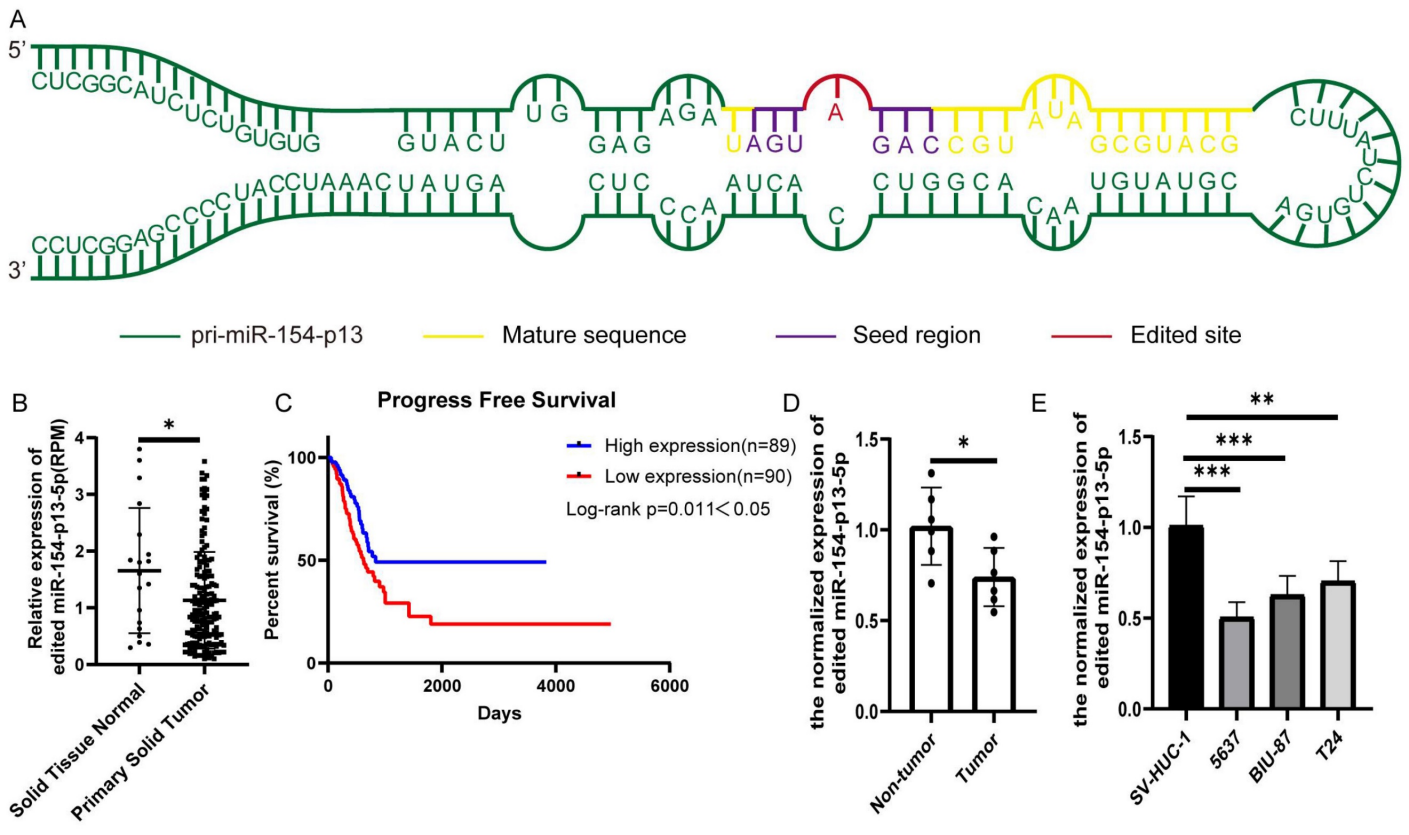
Edited miR-154-p13-5p expression was down-regulated in BC. (A) Schematic diagram of pri-miR-154-p13, illustrating the A-to-I miRNA editing site, with the mature sequence (orange), seed region (purple), and edited site (red). (B) miRNA-sequencing data from TCGA depicting the expression levels of edited miR-154-p13-5p in normal bladder and bladder cancer tissue samples. Raw expression levels of edited miR-154-p13-5p (reads per million mapped reads to miRNA [RPM]) were subjected to Mann-Whitney non-parametric comparison test. (C) The association between edited miR-154-p13-5p expression level and survival time in bladder cancer cases were collected from TCGA, with corresponding p-values based on the Mantel-Cox test. (D) Expression of edited miR-154-p13-5p in bladder cancer tissue samples, and para-cancerous tissue samples, as determined by RT-qPCR. (E) Expression of edited miR-154-p13-5p in normal bladder cells and bladder cancer cell lines, assessed via RT-qPCR.

To evaluate the biological significance of A-to-I edited miR-154-p13-5p in BC, we first identified miR-154-p13 editing hotspots through sequencing (Figure 1A). We compared different editing patterns between normal bladder and BC, and TCGA miRNA-sequencing data vividly showed edited miR-154-p13-5p expression in BC was lower than in normal bladder (Figure 1B). Interestingly, higher expression of edited miR-154-p13-5p in BC was significantly associated with better survival times (Figure 1C). The results from qRT-PCR in both bladder cell lines and tissue samples demonstrated that edited miR-154-p13-5p was down-regulated in BC (Figure 1D, 1E).

### Edited miR-154-p13-5p inhibited the proliferation and migration of BC cells

**Figure 2:**
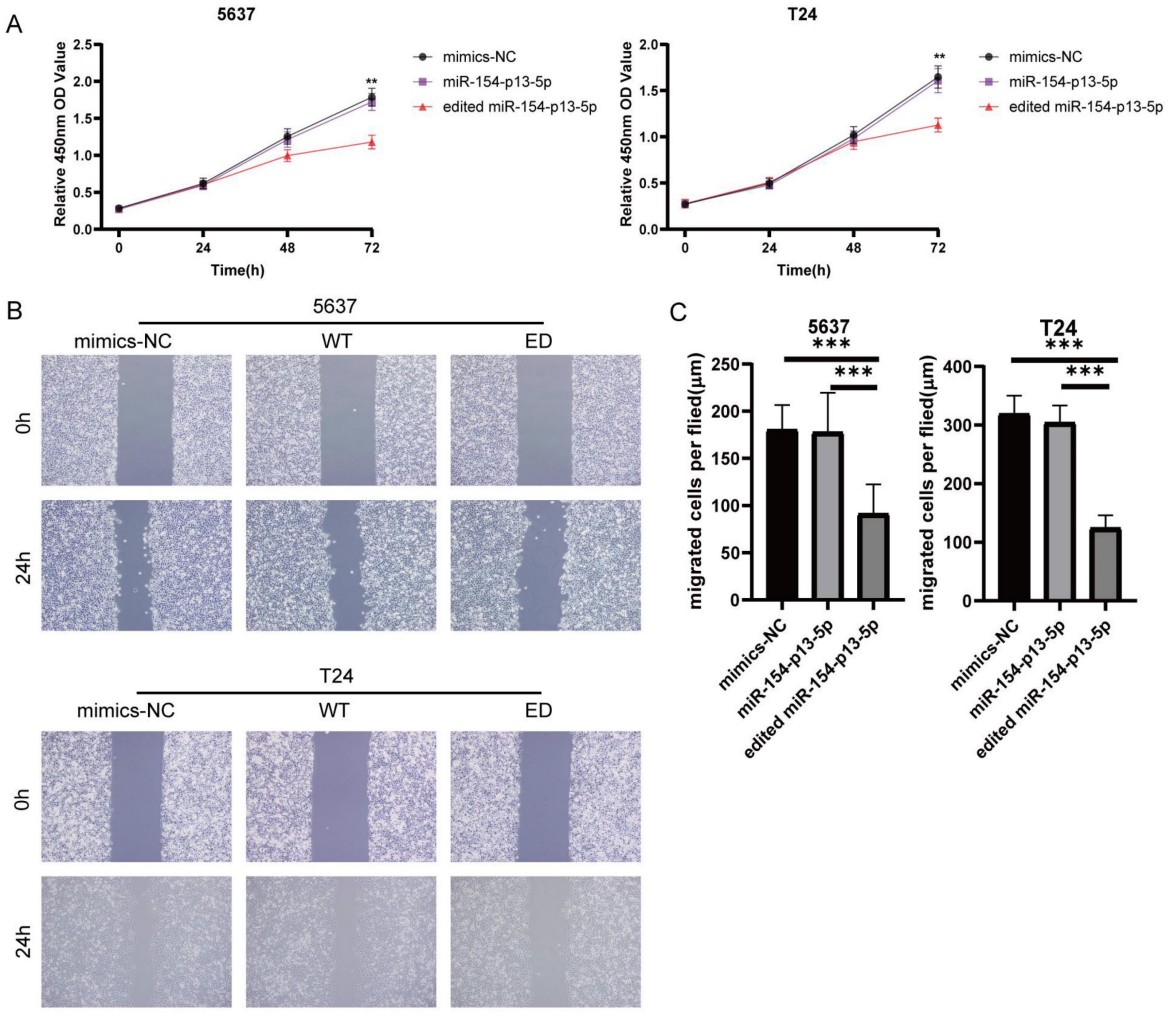
Edited miR-154-p13-5p inhibited the proliferation and migration of BC cells. (A) Assessment of influence exerted by edited miR-154-p13-5p on cell proliferation, using the CCK-8 assay. (B, C) Evaluation of cell migration distance over a 24h period, using the wound healing assay, following transfection with mimics-NC, miR-154-p13-5p (WT), and the edited one (ED).

To estimate the effect of edited miR-154-p13-5p in BC, we transfected the relevant miRNA mimics into cells. The findings from the CCK-8 assay revealed that miR-154-p13-5p had no discernible effect on cell proliferation. Oppositely, edited miR-154-p13-5p significantly suppressed cell proliferation (Figure 2A).

To investigate the effect of edited miR-154-p13-5p on cell motility, we conducted a wound healing assay to measure the distance of cell migration between 0 and 24 hours. The results showed migration distance of cells transfected with edited miR-154-p13-5p was significantly shorter than that of miR-154-p13-5p and mimics-NC, while miR-154-p13-5p had no significant difference compared to mimics-NC (Figure 2B, 2C).

### Edited miR-154-p13-5p induced apoptosis in BC cells

**Figure 3:**
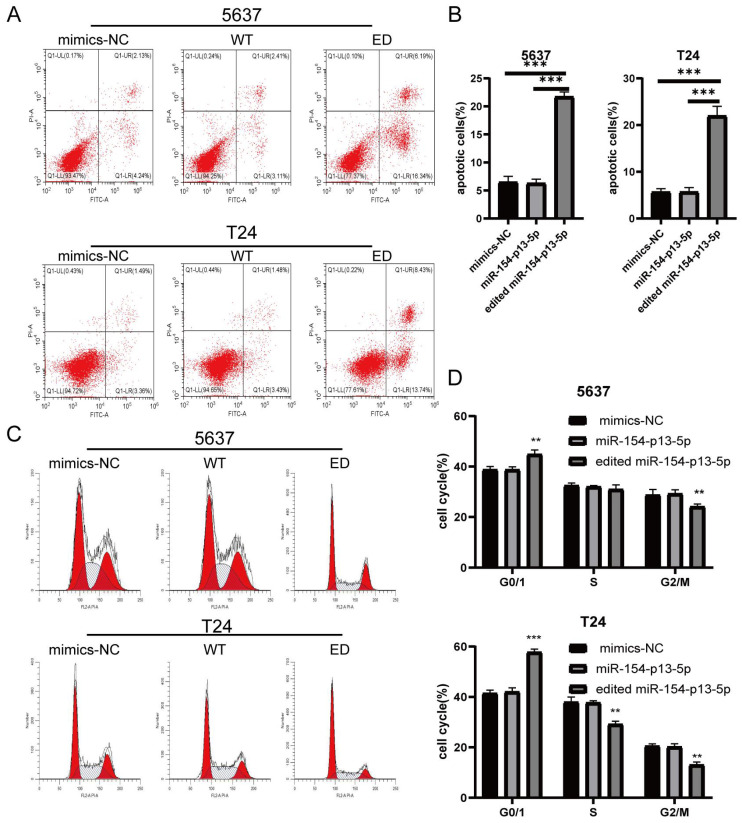
Edited miR-154-p13-5p induced apoptosis in BC cells. (A) Schematic representation of AV-FITC/PI-stained cells, illustrating early-apoptotic AV+ PI- and late-apoptotic AV+ PI+ cells. (B) Comparative analysis of the percentages of early and late apoptotic cells after transfection with mimics-NC, miR-154-p13-5p, and the edited one. (C) Cell cycle distribution of T24 and 5637 cells following transfection with related miRNA mimics. (D) Calculation and analysis of the percentages of cells in the G0/1, S, and G2/M phases.

To further elucidate the effects of edited miR-154-p13-5p on cell apoptosis, we conducted flow cytometry analysis to quantitatively evaluate early- and late-stage cell apoptosis utilizing Annexin V-FITC and PI staining. We explored their role in promoting cell apoptosis by calculating the overall percentage of apoptotic cells, which revealed a substantial increase in the percentage of apoptotic cell following the transfection of edited miR-154-p13-5p (Figure 3A, 3B).

We subsequently investigated the impact of edited miR-154-p13-5p on cell cycle disruption, for the induction of apoptosis could be mediated through cell cycle arrest. We investigated their influence on cell cycle distribution using flow cytometry. The results revealed a significant increase in the percentage of cells in the G0/G1 phase following transfection with edited miR-154-p13-5p, accompanied by a notable decrease in the G2/M phase (Figure 3C, 3D). The results obtained from the flow cytometry analysis and cell proliferation assays support our initial hypotheses, indicating that the modified miR-154-p13-5p may function as a tumor suppressor, impeding tumor progression.

### Analysis of differential expression genes (DEGs)

**Figure 4:**
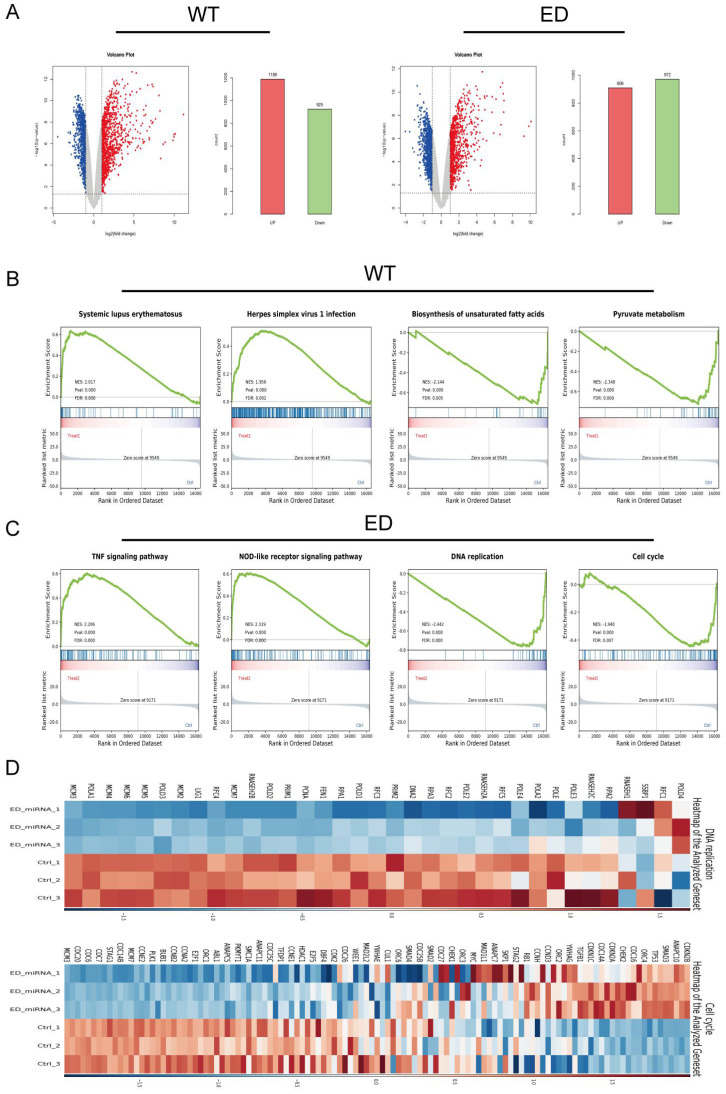
Analysis of differential expression genes (DEGs). (A) WT: Genes exhibiting up-regulation and down-regulation following miR-154-p13-5p transfection; ED: Genes showing up-regulation and down-regulation after edited miR-154-p13-5p transfection. (B, C) Gene Set Enrichment Analysis (GSEA) illustrating the enriched pathways for miR-154-p13-5p and the edited one. (D) Heatmap of differential genes enriched in DNA replication and cell cycle.

In our analysis, we adopted stringent criteria, defining genes as differentially expressed when they exhibited an absolute log FC >1 and a p-value < 0.05. To visualize the expression patterns of these genes, we generated volcano plots that distinctly highlight the DEGs. Our mRNA-sequencing findings unveiled that, compared with the control, 1189 elevated genes, and 925 suppressed genes following miR-154-p13-5p transfection. Similarly, 909 genes got elevated, and 972 genes got suppressed upon transfection of edited miR-154-p13-5p (Figure 4A). To delve deeper into the influence of miR-154-p13-5p editing events on biological pathways, we conducted a comprehensive Gene Set Enrichment Analysis (GSEA) using the pool of DEG resulting from miRNA transfection. The GSEA outcomes unveiled that the genes differentially expressed after miR-154-p13-5p transfection exhibited a positive correlation with pathways associated with Systemic lupus erythematosus and Herpes simplex virus 1 infection. Conversely, these genes displayed a negative correlation with pathways linked to biosynthesis of unsaturated fatty acids and Pyruvate metabolism (Figure 4B). Furthermore, the genes showing differential expression as a result of A-to-I edited miR-154-p13-5p transfection exhibited positive correlations with pathways such as the TNF signaling, NOD-like receptor signaling. Conversely, they demonstrated negative correlations with pathways involved in DNA replication, the Cell cycle (Figure 4C). To provide a more vivid representation of the GESA for DNA replication and the Cell cycle, we constructed a heatmap (Figure 4D).

### LIX1L served as the target of edited miR-154-p13-5p and presented to be a risky biomarker in BC

**Figure 5:**
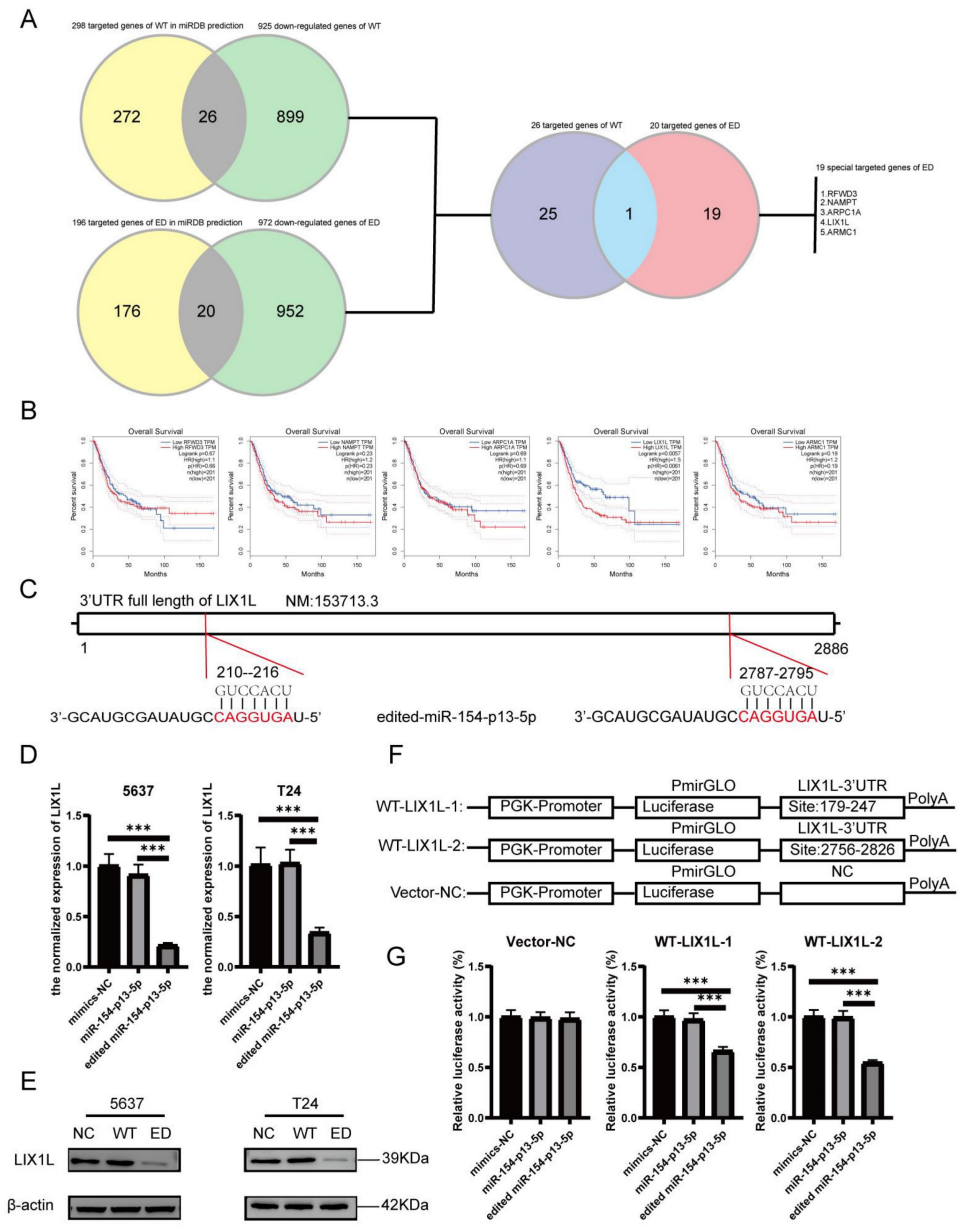
LIX1L served as the target of edited miR-154-p13-5p and presented to be a risky biomarker in BC. (A) Downstream targets of miRNA were identified among the down-regulated genes using miRDB database and mRNA-Seq. (B) Overall survival analysis of the remaining target genes in bladder cancer. (C) Schematic representation of LIX1L's 3'UTR full length and the binding sites of edited miR-154-p13-5p. (D) qPCR results indicating the expression levels of LIX1L in each group after transfection. (E)western blot results showing the differnt expression level of LIX1L after miRNA transfection. (F) Schematic diagram of pmirGLO Vector. (G) The result of Dual luciferase showing edited miR-154-p13-5p binding to two sites on the LIX1L 3'UTR.

To delineate the variances in cellular responses arising from the single nucleotide modification within miR-154-p13-5p, our primary focus was on the set of genes displaying down-regulation following diverse treatments. Employing the MiRDB database, we harnessed the mature miRNA sequence for target prediction, with the objective of identifying novel targets 27, 28. To achieve this, we cross-referenced the genes exhibiting down-regulation as per mRNA-seq data with the targets foreseen by miRDB and subsequently isolated common targets. Notably, we excluded targets already established as being regulated by miR-154-p13-5p (Figure 5A). After eliminating genes exhibiting undetectable baseline expression levels (RPKM>1), we embarked on survival analysis using GEPIA database 29 for the remaining genes. Remarkably, the survival analysis unveiled a strong association between elevated LIX1L expression and poorer survival outcomes (Figure 5B). This observation suggests that edited miR-154-p13-5p might curtail cell proliferation, migration and induce apoptosis by suppressing LIX1L expression. Moreover, guided by the sequence of edited miR-154-p13-5p, we pinpointed two binding sites within the 3′-UTR of LIX1L (Figure 5C). To validate LIX1L as a bona fide target of edited miR-154-p13-5p, we executed a battery of experiments, including RT-qPCR (Figure 5D) and western blot analyses (Figure 5E). These investigations unequivocally established that edited miR-154-p13-5p significantly impeded LIX1L expression at both the mRNA and protein levels, a phenomenon not observed in the case of miR-154-p13-5p. In addition, we conducted separately a dual-luciferase reporter assay, whereby plasmids harboring the two specific binding sites and a negative control were constructed and co-transfected with miRNAs into cells (Figure 5F). The outcomes clearly indicated that edited miR-154-p13-5p had a pronounced affinity for the two sites within LIX1L's 3'UTR, resulting in a significant reduction in luciferase activity, a characteristic not shared by miR-154-p13-5p (Figure 5G).

### The silence of LIX1L inhibited the proliferation and migration of BC cell

**Figure 6:**
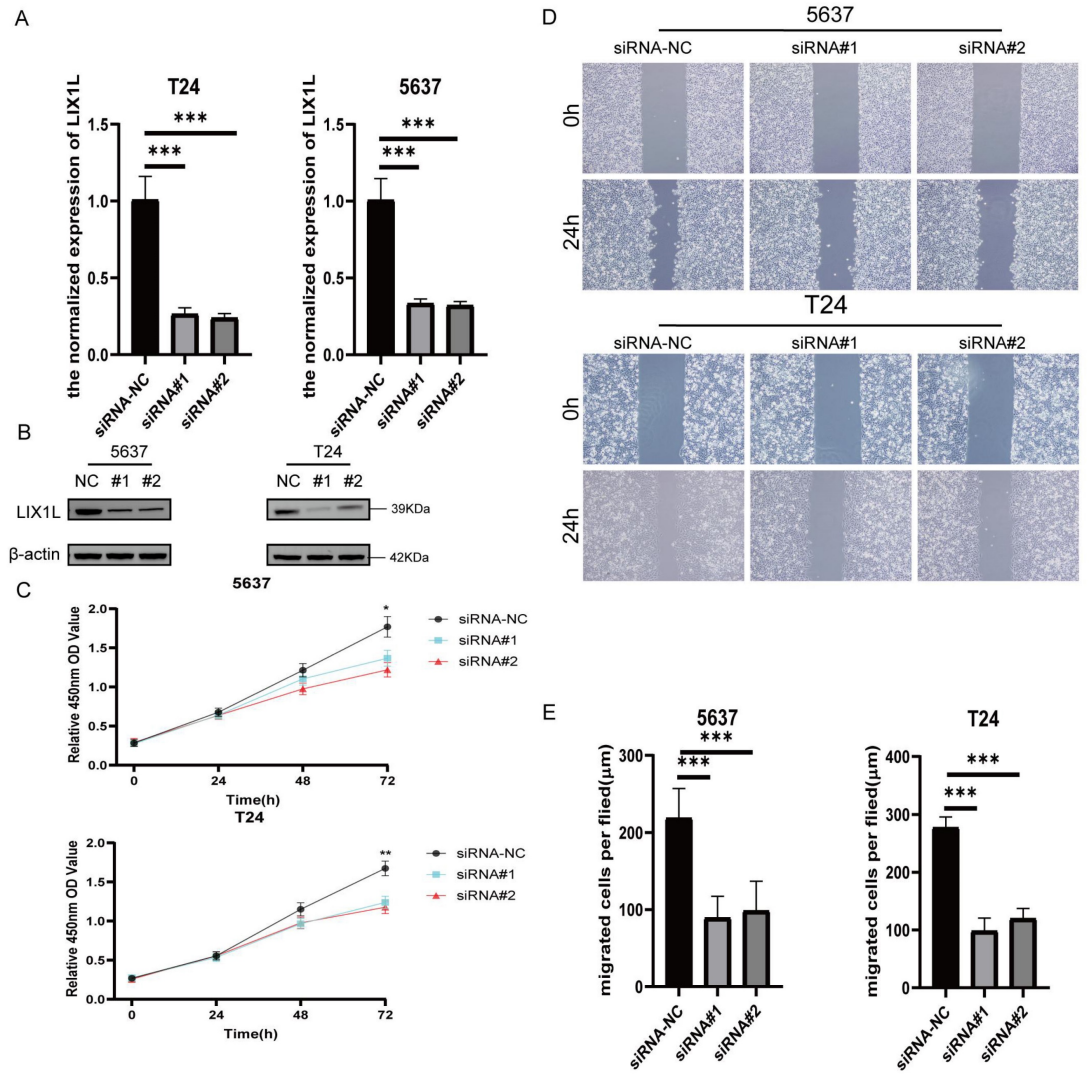
The silence of LIX1L inhibited the proliferation and migration of BC cell. (A, B) The silencing effect of siRNAs on LIX1L was confirmed by qPCR and western blot. (C) Cell proliferation was assessed using the CCK-8 assay to examine the effect of siRNAs. (D, E) The effect of siRNAs on cell migration was determined using the wound healing assay.

To investigate whether the phenotypic effects of edited miR-154-p13-5p are mediated through the silencing of LIX1L, we synthesized three LIX1L-specific siRNAs to target LIX1L. Initially, we validated that the first two siRNAs were effective in significantly inhibiting LIX1L expression at the mRNA level (Figure 6A). Furthermore, the first two siRNAs were shown to successfully silence the protein expression of LIX1L, as evidenced by western blot assays (Figure 6B). We then transfected these siRNAs to assess their impact on cell proliferation and migration ability. In CCK-8 assays, siRNA#1 and siRNA#2 were found to notably suppress proliferation of BC cells (Figure 6C). In the wound healing assay, both siRNA#1 and siRNA#2 significantly inhibited cell migration (Figure 6D, 6E).

### The silence of LIX1L induced apoptosis in BC cell

**Figure 7:**
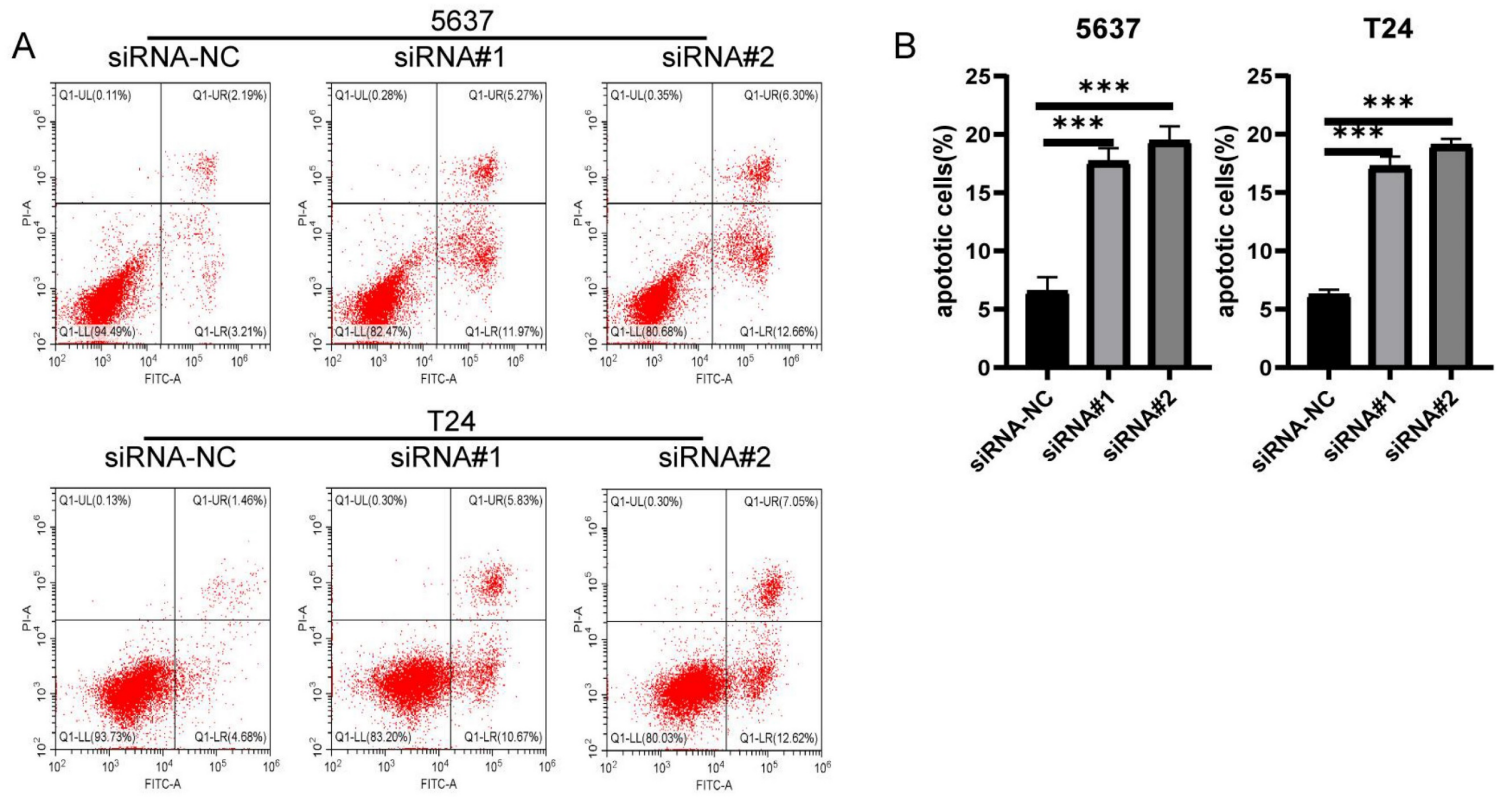
The silence of LIX1L induced apoptosis in BC cell. (A) The effect of siRNAs on cell apoptosis was evaluated using flow cytometry. (B) Statistical analysis of the percentage of apoptotic cells.

The results from flow cytometry indicated that siRNA#1 and siRNA#2 promoted cell apoptosis by silencing LIX1L, aligning with the phenotypic influences of edited miR-154-p13-5p (Figure 7A, 7B).

### Edited miR-154-p13-5p downregulated CDK2 and up-regulating cleaved caspase-3 expression by targeting LIX1L in BC cell

**Figure 8:**
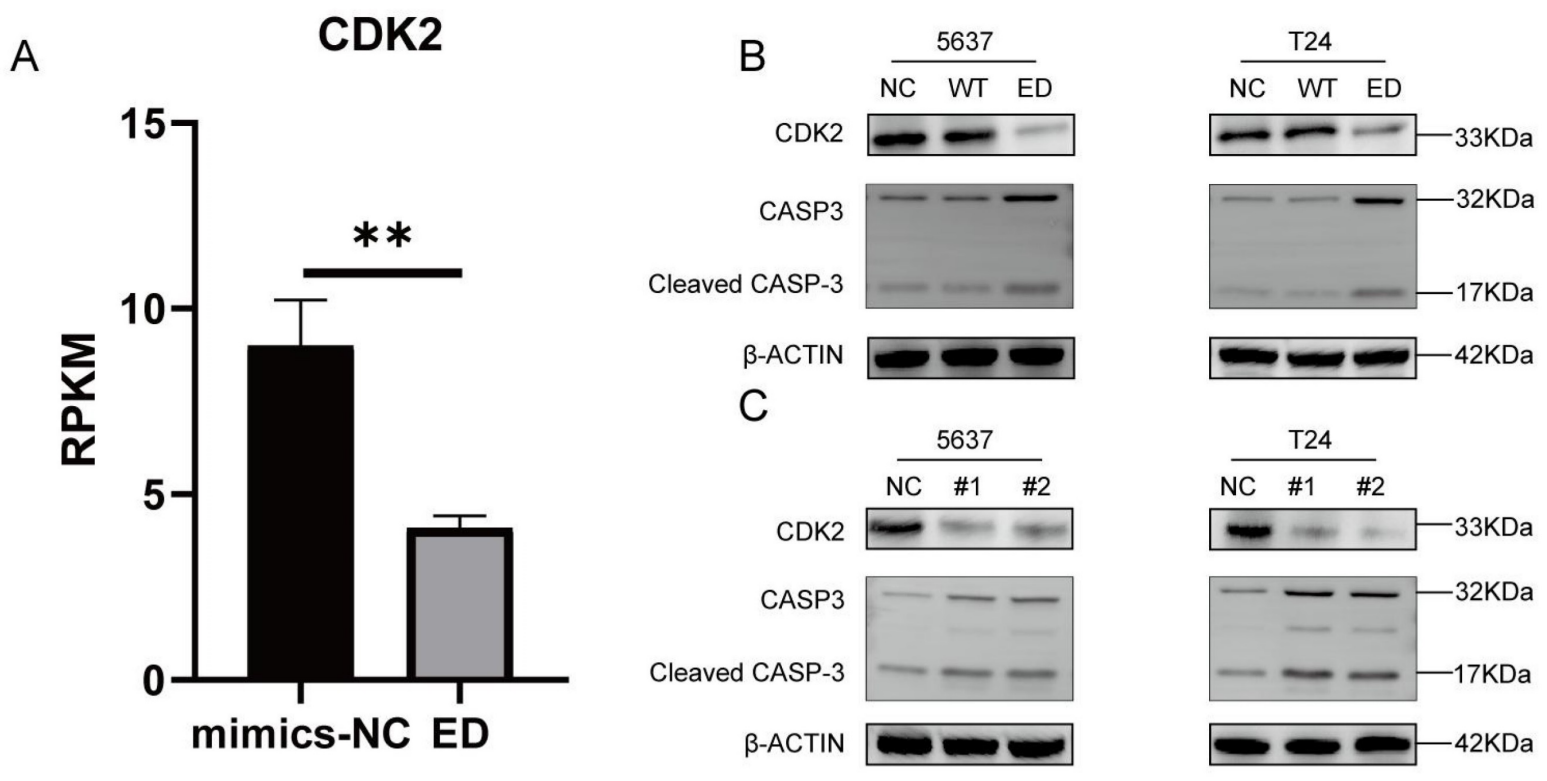
Edited miR-154-p13-5p downregulated CDK2 and up-regulating cleaved caspase-3 expression by targeting LIX1L in BC cell. (A) Presents the mRNA-Seq analysis of CDK2 expression levels post-transfection with edited miR-154-p13-5p in BC cells. (B, C) Evaluation of the impact of related miRNA and siRNAs on the expression levels of CDK2 and cleaved caspase-3 and caspase-3. #1 = siRNA#1, #2 = siRNA#2.

In different stages, various cyclins regulate the cell cycle, among which cyclin A and cyclin E activate CDK2 to control chromosome replication 30. In mRNA-seq, we observed a downregulation of CDK2 expression after transfection with edited miR-154-p13-5p (Figure 8A). To further validate the regulatory effects of edited miR-154-p13-5p and LIX1L-siRNAs on CDK2, Western blot analysis revealed a downregulation of CDK2 expression after transfection with miR-154-p13-5p and siRNAs (Figure 8B, 8C).

One of the most important characteristics of tumor is the avoidance of apoptosis, so the initiation of tumor cell apoptosis is considered to be an important biological event, in which cysteine-dependent aspart-specific proteases play an important role, especially caspase-3^31^, ^32^. During the intricate cascade of cell apoptosis, caspase-3 is initially activated, leading to the cleavage of its substrates, DNA fragmentation, and ultimately, cell apoptosis 33-35. To explore the relationship between these observations, we transfected the corresponding siRNAs and miRNAs separately and conducted a western blot assay. The outcomes unequivocally unveiled a significant upregulation in the expression of cleaved caspase-3 within cells subjected to edited miR-154-p13-5p, siRNA#1, and siRNA#2 transfections. This suggests that edited miR-154-p13-5p promotes apoptosis in BC cells by suppressing LIX1L (Figure 8B, 8C).

### Edited miR-154-p13-5p inhibits the EMT-related proteins VIM and FN1 in BC cells by targeting LIX1L

**Figure 9:**
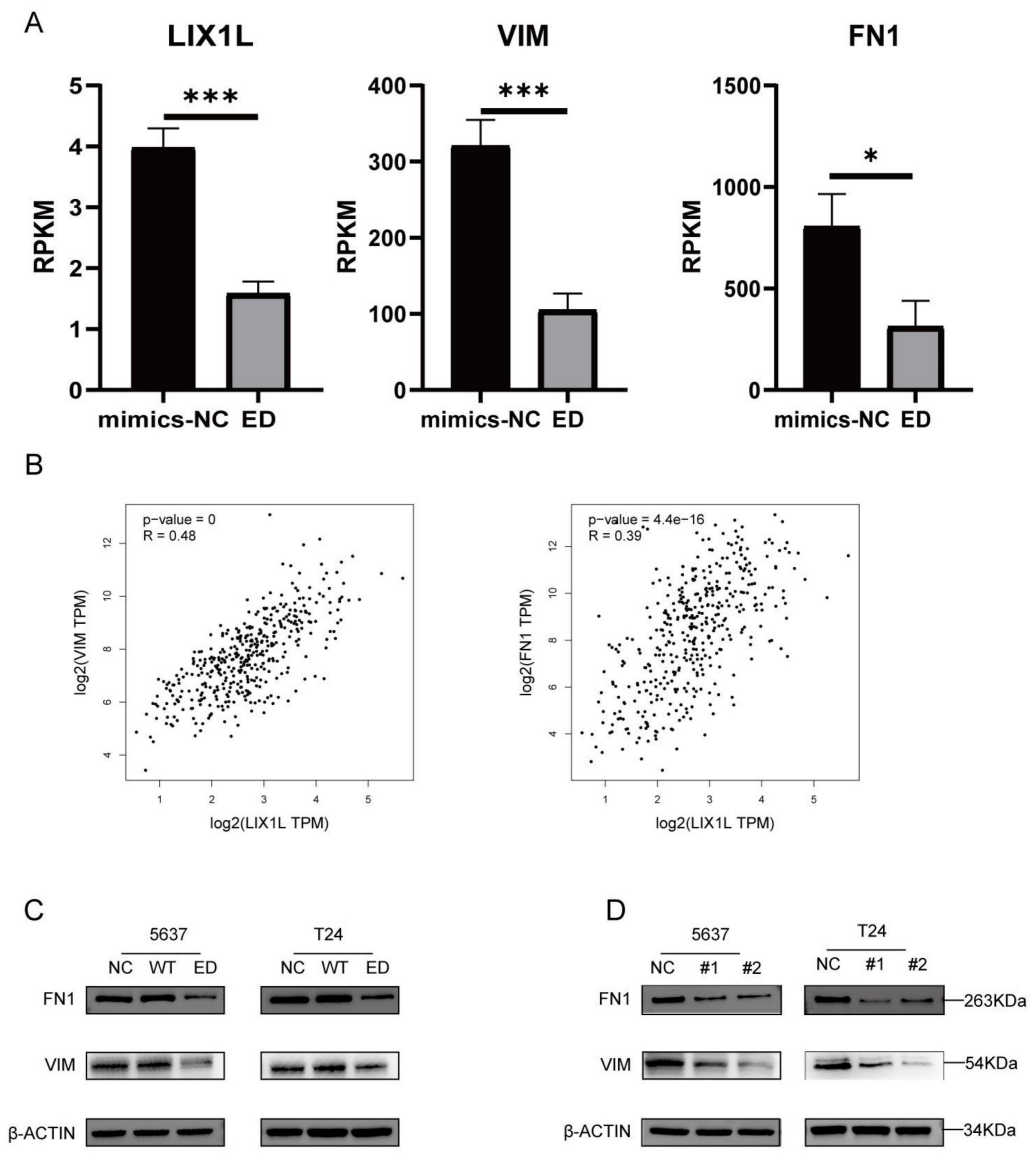
Edited miR-154-p13-5p inhibits the EMT-related proteins VIM and FN1 in BC cells by targeting LIX1L. (A) Analysis of mRNA-Seq datas after transfecting with edited miR-154-p13-5p. (B) Pearson correlation analysis of LIX1L and EMT related proteins (VIM and FN1) expression in bladder cancer in TCGA database. (C, D) The results of Western blot assays showing the regulatory relationship between LIX1L with EMT relative protein (VIM and FN1) after transfecting with miRNAs or siRNAs.

Through mRNA-seq analysis, we observed a downregulation in the expression of LIX1L, Vimentin (VIM), and Fibronectin 1 (FN1) (Figure 9A). To elucidate the correlation between LIX1L and the expressions of VIM and FN1, we conducted Pearson correlation analysis (Figure 9B). Based on the correlation coefficient grading, LIX1L exhibited a moderate correlation with the expressions of VIM and FN1 36. To further validate the regulatory relationship among them, we separately transfected miRNAs and LIX1L-siRNAs. The results indicated a downregulation in both VIM and FN1 expressions. This suggests that the expressions of VIM and FN1 are regulated by edited miR-154-p13-5p and LIX1L-siRNAs, while they are not regulated by miR-154-p13-5p (Figure 9C, 9D).

## Discussion

As research on miRNA editing advances, an increasing number of RNA editing sites are being uncovered within the human transcriptome. Currently, 2 RNA editing types are recognized, encompassing A-to-I and C-to-U editing, with A-to-I editing being predominant 37. A-to-I miRNA editing events appear to exhibit different editing patterns between normal tissue and its corresponding tumor tissue. In various normal tissues like breast, liver, and lung, and their associated tumor tissues, 11 A-to-I editing sites have been identified within the seed region of mature miRNAs. Among these, 4 A-to-I edited miRNAs exhibit upregulated expression in tumor tissues, while the remaining 7 A-to-I edited miRNAs display downregulated expression in tumor tissues 12. However, there is a tendency for miRNA editing levels to be relatively higher in normal tissues compared to tumors. Analysis of 10,593 miRNA-seq samples from TCGA, covering 32 different cancer types and normal tissues, revealed significantly lower editing levels in 19 tumor samples compared to normal tissue 38.

Changes in a single base in the seed region of mature miRNA can cause changes in a set of targets 39, 40. Moreover, the A-to-I edited miRNAs appear to be more likely to bind to oncogenes. 8 A-to-I edited miRNAs were forecasted to bind new targets involved in cell proliferation process, growth, and survival function 38. Interestingly, miRNA editing has a tendency to suppress tumor processes. For instance, within various tumor categories including Breast Cancer, Ovarian Cancer, Kidney Cancer and Lung Cancer, edited miR-379-5p demonstrates the capability to repress cell proliferation and provoke apoptosis through its interaction with CD97 among the novel target genes 41. In the context of de novo glioblastoma, a highly aggressive brain cancer, ADAR2-mediated editing of miR-589-3p results in a shift from its regulation of the tumor suppressor PCDH9 to the modulation of ADAM12, effectively impeding glioblastoma invasion 42.

In our previous investigation, we highlighted A-to-I RNA editing levels within miR-154-p13-5p in bladder cancer, particularly in comparison to other edited miRNAs, and demonstrated an intriguing connection between the editing patterns of the miR-154 family and the prognosis of bladder cancer 43. Despite these promising insights, the underlying mechanisms linking miR-154 family editing and bladder cancer prognosis remain enigmatic, necessitating further in-depth exploration. Furthermore, it was documented that the ADAR1 enzyme catalyzes A-to-I editing of miR-154-p13-5p, resulting in the conversion of a single base of its mature sequence 19. Notably, the miRNA-sequencing analysis of biological large samples unveiled a noteworthy reduction in miR-154-p13 editing levels in BC tissues in comparison to the normal 38.

In the present study, we unveiled a pivotal discovery concerning the inhibitory role of edited miR-154-p13-5p in BC by significantly curbing cell proliferation, migration, and instigating apoptosis, primarily through its precise modulation of LIX1L. Firstly, we confirmed the low expression of edited miR-154-p13-5p in bladder cancer by miRNA sequencing data in TCGA and RT-qPCR assays results. Secondly, we screened edited miR-154-p13-5p key target genes by mRNA-Seq and survival analysis. Finally, we found the regulatory relationship between LIX1L and CDK2, Caspase-3, Cleaved caspase-3 and EMT relative genes by Western blotting assays.

Limb and CNS expressed 1 like (LIX1L) was initially identified during early chicken limb development and subsequently detected as commonly expressed in the in human tumor tissues such as gastric, colon and lung cancers 44, 45. LIX1L is an established RNA-binding protein (RBP) that acts as pivotal determinant within post-transcriptional regulation. It features a double-stranded RNA (dsRNA) binding motif, facilitating its interaction with specific microRNAs and mRNA molecules, thereby modulating their expression. During chronic liver injury, LIX1L activates the hepatic fibrosis process through the interaction of CCL20 mRNA 46. In the liver cancer, LIX1L interacts with miR-21-3p, upregulating its expression, which in turn targets and suppresses FBP1 expression, ultimately enhancing glucose consumption and lactic acid production 47. TATDN1 (lncRNA) indirectly upregulates LIX1L by targeting and down-regulating miRNA-6089 expression, thereby promoting cell proliferation and cell cycle progression 48. These studies collectively underscore the significant role of LIX1L in disease progression, justifying its selection as the focus of our further investigations.

Epithelial-to-Mesenchymal Transition (EMT) is a crucial biological process in which malignant tumor cells of epithelial origin lose cell polarity and epithelial characteristics, leading to enhanced migration and invasion capabilities by attaching to the basement membrane and acquiring mesenchymal phenotypes 49. Interestingly, the downregulation of epithelial cell markers serves as a hallmark of EMT, like cytokeratins and E-cadherin, and so does the upregulation of mesenchymal cell markers, like N-cadherin, vimentin, and FN1 50. LIX1L has been proposed as a novel marker associated with mesenchymal characteristics and closely linked to the process of EMT 51, 52. The relationship between LIX1L and EMT-related protein set remained unclear in BC, and our study offered a solution. We analyzed the relationship by combing Pearson correlation with mRNA-Seq analysis, and confirmed it through a western blot assay in which their expressions were positively correlated.

Cyclin-dependent kinases (Cdks) are a class of serine/threonine kinases that serve as crucial regulatory enzymes driving all cell cycle transitions 53. After the cyclin E/CDK2 complex completes phosphorylation of the retinoblastoma protein (RB), it drives the G1/S transition. Subsequently, during the S phase, CDK2 regulates DNA replication and centrosome duplication 54, 55. Currently, an increasing amount of research indicates that inducing a downregulation of CDK2 expression through siRNA technology triggers DNA repair, apoptosis and cell cycle arrest 56. In this study, our findings indicate that transfection with edited miR-154-p13-5p or LIX1L-siRNAs led to cell cycle arrest in the G1 phase, accompanied by a reduction in CDK2 expression levels.

The knockout of LIX1L has been reported to inhibit cell viability and concomitantly trigger an elevation in caspase-9 and caspase-3/7 activity in gastric cancer, as assessed by ELISA assay 45. However, the precise mechanistic link between LIX1L and cell apoptosis remains unclear. Cleaved caspase-3 is consistently recognized as a dependable marker for cell apoptosis. For instance, a study examining the molecular mechanism by which paclitaxel induces apoptosis revealed that when human breast cancer cells were treated with paclitaxel, the levels of uncut Caspase-3 significantly decreased. However, western blots illustrated an increase in the levels of cleaved caspase-3 57. Our investigation confirmed that the silencing of LIX1L led to a significant increase in the percentage of apoptotic cells, as assessed by flow cytometry. Additionally, the expression of cleaved caspase-3, a recognized strong indicator of cell death induction, displayed a significant increase.

Overall, there are certain limitations to our study. Although we investigated apoptosis induction and downregulation of CDK2 expression, along with upregulation of Cleaved Caspase-3 expression in bladder cancer cells transfected with edited miR-154-p13-5p or LIX1L-siRNAs, we did not further elucidate the direct connection between CDK2 and Cleaved Caspase-3. Furthermore, our study demonstrated the potential of edited miR-154-p13-5p to improve the prognosis of BC *in vitro*, but further research is required to confirm its efficacy in an *in vivo* setting.

## Conclusion

In conclusion, our studies have revealed that edited miR-154-p13-5p significantly inhibits cell proliferation and migration while inducing apoptosis. Through mRNA-sequencing, we have identified potential downstream targets of edited miR-154-p13-5p. Furthermore, we employed survival analysis in conjunction with the miRDB database to predict that these phenotypic effects may be attributed to the silencing of LIX1L among the downstream targets. PCR, western blot assays, and luciferase assays have consistently demonstrated that LIX1L is a downstream target of edited miR-154-p13-5p, rather than miR-154-p13-5p itself. Subsequent investigations revealed that LIX1L interacted with EMT, CDK2, and Cleaved Caspase-3. Collectively, our findings provide a new perspective, indicating that increasing the expression of edited miR-154-p13-5p may be a promising strategy for impeding the advancement of BC.

## Figures and Tables

**Table 1:** BC patient information

Patients	Age	Gender	Tissue	Pathologic tumor staging	Gender	Treatment
No.1	65	F	T	MIBC	HG	LRC
			P	Non-tumor		
No.2	78	M	T	MIBC	LG	LRC
			P	Non-tumor		
No.3	70	M	T	MIBC	HG	LRC
			P	Non-tumor		
No.4	78	F	T	MIBC	HG	LRC
			P	Non-tumor		
No.5	66	M	T	MIBC	HG	LRC
			P	Non-tumor		
No.6	69	M	T	MIBC	HG	LRC
			P	Non-tumor		

BC = Bladder Cancer. Gender, F = Female, M = Male. P = Precancerous tissue, less than 3cm away from the tumor tissue, T = Tumor. MIBC = Muscle-invasive bladder cancer. LG = Low pathological grade, HG = High pathological grade. LRC = Laparoscopic radical cystectomy.

**Table 2:** miRNA and siRNA

Mimics	Sequence (5'-3')
mimics-NC	UUUGUACUACACAAAAGUACUG(Sense)
	CAGUACUUUUGUGUAGUACAAA(Antisense)
miR-154-p13-5p	UAGUAGACCGUAUAGCGUACG(Sense)
	CGUACGCUAUACGGUCUACUA(Antisense)
edited miR-154-p13-5p	UAGUGGACCGUAUAGCGUACG(Sense)
	CGUACGCUAUACGGUCCACUA(Antisense)
SiRNA-NC	UUCUCCGAACGUGUCACGUTT(Sense)
	ACGUGACACGUUCGGAGAATT(Antisense)
SiRNA#1	GGGCUAUGGCCGAGUGAAUTT(Sense)
	AUUCACUCGGCCAUAGCCCUTT(Antisense)
SiRNA#2	GCUAAUGAAUUCUGUGUUUTT(Sense)
	AAACACAGAAUUCAUUAGCTT(Antisense)
SiRNA#3	GCAAAUCAAUGUUGGAGUUTT(Sense)
	AACUCCAACAUUGAUUUGCTT(Antisense)

## Abbreviations

TCGA: The Cancer Genome Atlas
BUC: Bladder urothelial carcinoma
LIX1L: Limb and CNS expressed 1 like
NF1: Neurofibromin 1
PI: Propidium iodide
FITC: Fluorescein Isothiocyanate
LRC: Laparoscopic radical cystectomy
PFS: Progression-Free Survival
PVDF: Polyvinylidene Difluoride
PMSF: phenylmethylsulfonyl fluorid
PIC: Protease Inhibitor Cocktail
